# Eye Selection Criteria’s Influence in the Value of Pituitary Macroadenoma Management Biomarkers: Preliminary Findings

**DOI:** 10.3390/jcm14134542

**Published:** 2025-06-26

**Authors:** Odelaisys Hernández-Echevarría, Elizabeth Bárbara Cuétara-Lugo, Mario Jesús Pérez-Benítez, Lídice Galán-García, Ibrain Piloto-Diaz, Eduardo Fernández

**Affiliations:** 1 Institute of Bioengineering, Universidad Miguel Hernández, 03202 Elche, Spain; 2 Cuban Institute of Ophthalmology “Ramón Pando Ferrer”, University of Medical Sciences of Havana, Havana 10400, Cuba; ecuetaral@gmail.com (E.B.C.-L.); marito1992jpb@gmail.com (M.J.P.-B.); ipdiaz@infomed.sld.cu (I.P.-D.); 3 Cuban Center for Neurosciences, Havana 11300, Cuba; lidicegalan2000@gmail.com

**Keywords:** eye selection criteria, pituitary macroadenoma, bi-nasal sectors, pattern visual evoked potentials, visual function recovery prediction

## Abstract

**Objectives**: To elucidate the influence of eye selection criteria (ESC) on the reliability of biomarkers in diagnosis and prediction using pre-surgical parameters, assessments were undertaken as the subject of analysis. **Methods**: Pituitary macroadenoma (PMA) diagnosis and postsurgical visual function recovery biomarker analysis was used as the subject to illustrate the point. Six datasets (right, left, best, worst, random and both eyes), derived from a longitudinal study that involved 42 PMA patients and age-matched healthy volunteers, were generated. A comparison of the diagnostic efficacies of the amplitude of pattern visual evoked potentials (pVEP) and bi-nasal sector thickness in the ganglion cells complex plus the inner plexiform layer was performed using ESC. Afterwards, multivariate models for PMA diagnosis and the prediction of postsurgical visual function recovery, using Stable Sparse Biomarkers Detection methodology, were developed. A comprehensive evaluation was performed once for controls and in pre-surgical PMA patients at 3 and 12 months after transsphenoidal tumor removal. **Results**: The proposed biomarkers displayed specificity and sensibility ≥ 0.74 and AUC ≥ 0.87. The diagnostic values derived were ESC-dependent. All the prediction models had accuracies over 0.96, and the proposed biomarkers had stability ≥ 99% and the highest β values. **Conclusions**: Although the diagnostic values of the proposed biomarkers are affected by ESC, they exhibit equal accuracy for the same eye. Worse eye data represent the best choice for the analysis. Further studies are needed to validate the models for use in the prediction of the 12-month postsurgical restoration of parvocellular traffic.

## 1. Introduction

In ophthalmology, the unit of analysis is usually the eye; therefore, having distinct data for two eyes represents a challenge in design, analysis and interpretation. Although this issue has already been discussed by several authors, it is not uncommon that well-planned studies (clinical trials) ignore it, and this frequently gives rise to statistical errors [1,2,3,4]. Therefore, eye selection criteria (ESC) remain a controversial issue in the vision research community [5]. The presentation of an overall summary of ocular findings from two eyes per individual, in a similar fashion to the use of only one eye per individual, may result in a surplus of information; this is wasteful, and may lead to imprecision.

Conversely, an analysis of individual eyes with no allowance made for inter-eye correlations may result in falsely narrow confidence intervals (CI) for the estimates of effects. Armstrong provided valuable advice regarding ESC in research design [6], namely, to collect data from both eyes so as to reduce the number of subjects that have to be recruited and potentially increase the study’s statistical power. The random selection of one eye when both are eligible, unless a specific choice can be justified, and considering the alternate eye as a control if one eye is chosen based on clinical criteria, are recommended. Inter-eye correlation calculations using the interclass correlation (ICC) should be taken into account for decision-making. The alternatives are to include both eyes if there is no correlation or to select one eye at random if the correlation is close to one.

Ophthalmic research involves the analysis of data from paired organs, namely, the eyes. Some diseases are unilateral, such as choroid melanoma [7] and Coats disease [8], while others are bilateral and can be symmetrical (blepharitis) [9] or not (senile cataract [10], open angle glaucoma [11] and inherited retinal diseases) [12,13]. Papilledema in the course of idiopathic intracranial hypertension is typically bilateral and symmetric, but it may also be asymmetric or unilateral [14]. The abovementioned scenarios illustrate the important of using appropriate ESC in ophthalmic research in order to arrive at accurate conclusions.

Some authors choose individuals as the unit of analysis, and for that purpose, they use modeling approaches that can accommodate correlated binary data, such as the Generalized Linear Mixed Model, which uses the maximum likelihood estimation, and the marginal model, which uses the Generalized Estimating Equation (GEE). The GEE is a general statistical approach used to fit a marginal model for the analysis of longitudinal and pooled data. It is useful for analyzing data collected in clusters when observations within a cluster may be correlated or observations in separate groups are independent, or for monotonous transformations [15]. Mathematical modeling can be helpful in complementing ESC in ophthalmic research, helping to understand ocular dynamics, reducing experimental costs and enhancing treatment strategies and optimizing study design (by assisting in selecting the most appropriate eye(s) for study through the analysis of factors like symmetry, dominance or disease progression and ensuring that the data collected are both relevant and statistically robust) and in predictive analytics (by incorporating patient-specific data, mathematical models can predict disease progression or treatment outcomes). The last two are addressed in the present study.

Many authors have addressed bitemporal hemianopia in the visual field as the main neuro-ophthalmologic finding in chiasm compression and optical coherence tomography (OCT), as well as pVEP parameters as surrogate variables, which depends on patient cooperation. In PMA, as a result of visual pathway compression, pVEP amplitudes can be affected by the nerve conduction blockade of the damaged fibers. In addition, no emphasis has previously been placed on the segmentation of the retinal ganglion cell structure. Previous work by our team has focused on introducing pattern visual evoked potential amplitudes and the thicknesses of bi-nasal sectors of the ganglion cells complex plus the inner plexiform layer (GCC+IPL) as biomarkers in the management of pituitary macroadenoma patients (PMA) [16], since there is no consensus on which biomarkers are the best choice for PMA management. We decided to perform a similar analysis using datasets generated with typical ESC. This project aimed at elucidating the influence of ESC on the diagnostic values of two PMA biomarkers and the predictive values of pre-surgical parameters taken together for assessing post-surgical visual function restoration.

## 2. Materials and Methods

Data were obtained from a longitudinal study that involved 42 patients diagnosed with PMA from the Neuro-ophthalmology Service of the Cuban Eye Institute from March 2017 to June 2021. The Institutional Review Bureau (#12/2017) and the Ethics Committee (#27/2017) approved this research in February 2021. Participants expressed their willingness to participate in the study by signing the informed consent form [16]. The research was conducted according to the principles delineated in the Helsinki Declaration 7th Brazil revision, 2013 (World Medical Association) [17].

### 2.1. Study Design

Data were generated in a study that involved two groups. The first comprised PMA patients with diagnoses confirmed by the Pathology Department of the National Minimal Access Center in Havana, where trans-sphenoidal surgery was performed. In the second group, healthy volunteers were evaluated at a single time. Patients were reassessed at three and twelve months after surgery. For data analysis, six datasets were generated according to the following criteria: right eye (RE), left eye (LE), randomly selected eye (RSE), both eyes (BoE), best eye (BeE) and worst eye (WE). Although best-corrected visual acuity loss and visual field defects have traditionally been used as criteria to identify best and worst eyes, these parameters could remain normal in the early stages of chiasmal compression, and their values could be subjective because they depend on patients’ input during the measurements. In addition, the global retinal nerve fiber layer (gRNFL) is a structural parameter that can be objectively and accurately determined by OCT. This is why gRNFL thinning was the criteria used in the present study. Thus, the worst eye was taken as the one with a gRNFL lower than the other. In the randomly selected eyes group, each patient contributed with data from only one eye, ensuring a subset that equaled the number of patients. The eye was chosen by a python code, selecting the record in the corresponding data frame of the right or left eye according to the value of a number generated by the random library; if the random number was between 0 and 0.5, a record was selected in one eye, and if it was between 0.5 and 1, the record was selected in the opposite eye. In the ‘both eyes’ group, each patient contributed data from both eyes separately in the form of independent observations whenever available.

The present study had two parts. First was the comparison of the PMA biomarkers’ diagnostic values according to typical ESC. The second part was focused on developing multivariate models for PMA diagnosis and 3 and 12 months’ post-surgical visual function recovery prediction.

### 2.2. Eligibility Criteria for Participants

Patients older than 18 years with suggestive PMA symptoms with magnetic resonance imaging (MRI) confirmation, who were able to be tested for best-corrected visual acuity up to 0.5 log MAR, automated visual fields and pVEP testing, were included. Data from both eyes were collected when available. The exclusion criteria were spherical refractive error outside over 5 diopters or greater than 2 diopters of astigmatism, unreliable preoperative visual field testing [18], anterior ophthalmologic segment disease, retina or optic nerve diseases and non-attendance of postsurgical evaluations.

### 2.3. Clinical and Neuro-Ophthalmological Examination

Clinical evaluation comprised a comprehensive neuro-ophthalmological examination, with best-corrected visual acuity (Bailey–Lovie log MAR chart, University of Melbourne, Parkville, Australia), color vision (CV, Ishihara 16 plates, Kanehara Shuppan Co., Ltd., Tokyo, Japan), contrast sensitivity (CS, Pelli-Robson, La Salle, IL, USA), intraocular pressure assessment by applanation tonometry (Haag Strait, Köniz, Switzerland), pupillary reflexes, confrontation visual field, ocular motility, Hertel ophthalmometry, anterior segment slit-lamp biomicroscopy and fundus examination using indirect binocular ophthalmoscopy with fully dilated pupils. Median deviation was assessed by Octopus 101 perimetry (32 programs, Dynamic strategy, Haag Strait, Switzerland) [18,19].

The latencies and amplitudes of the P100 components of pVEP were obtained with the RETI-port/scan system (Roland Consult, Brandenburg an der Havel, Germany). Recordings were obtained monocularly according to the International Society for Clinical Electrophysiology of Vision standards [20,21,22]. MRI sequences of axial, coronal and sagittal slices at 3 mm from T1, T2, FLAIR and STIR sequences of the brain and orbit with and without gadolinium intravenous contrast were performed in all patients to detect the tumors, as described elsewhere [23,24].

Global retinal nerve fiber layer and ganglion cell complex plus inner plexiform layer (gGCC+IPL) thicknesses (µm) were assessed using two automatic segmentation algorithms. Optical coherence tomography was performed with a Cirrus-5000 (Carl Zeiss Meditec, Dublin, CA, USA). Each eye was scanned three times using macular cube (512 × 128-line scans) and optic disk cube (200-line scans) protocols. Records with signal strength above six arbitrary units of 6 mm cube scanned areas without signal averaging were used in the analysis.

### 2.4. Sample Description

The patients with pituitary macroadenomas were identified between March 2017 and June 2021 at the Cuban Institute of Ophthalmology, and 42 met the inclusion criteria after excluding cases with ophthalmological comorbidities, advanced disease precluding testing or alternative diagnoses. The 42 PMA patients selected for the study were age-matched with 42 healthy volunteers. The Match It library in R (4.1.2), which contains the script for Propensity Score Matching, was used for this purpose, applied to an initial healthy individual dataset that included 65 subjects. For the healthy volunteers’ group, one eye per person was randomly selected with the same method as was used to choose RSE.

### 2.5. Missing Data

Linear regression with a standard deviation perturbation was the imputation method used for the single imputation of electrophysiological missing data from bi-nasal sectors of GCC+IPL thickness data using SciPy (1.11.1). Stats (0.14.0), a statistical module in Python (3.11.5). When OCT data were missing, the five nearest neighbors were used.

### 2.6. General Methods of Data Analysis

Normality was assessed using the Kolmogorov–Smirnov test or Shapiro–Wilks (depending on data size), and homoscedasticity was verified using the Levene test. To compare media among datasets, the Kruskal–Wallis test was used with the Holm adjusted Wilcoxon test. Inter-eye correlation was explored. Inter-class correlation was calculated based on the Pearson correlation statistic, using pingouin (0.5.4), an intraclass_corr function of Python. Koo Li criteria were used to qualify the magnitude of the ICC [25].

### 2.7. Diagnostic Precision Analysis

Biomarkers’ accuracies were estimated using two basic measures, sensitivity and specificity, derived from the values in the confusion matrix of the parameters of interest. The continuous parameters of interest were dichotomized using cut-off points—15.5 µV for pVEP amplitude in Oz at 12′ (AOz12′) and 65 µm for bi-nasal sectors. Formulae for sensitivity (Se) and specificity (Sp) are described elsewhere (Se = $\frac{number\;of\;true\;positive}{total\;of\;individual\;with\;the\;illiness}$ and Sp = $\frac{number\;of\;true\;negative}{total\;of\;individual\;without\;the\;illiness}$). In addition, receiver operator characteristics (ROC) curves were obtained by plotting Se vs. 1-Sp with the NumPy package (1.24.3), and area under the curve (AUC) was calculated and compared using nonparametric testing based on permutations of the z statistic. Bootstrap analysis was conducted with 10,000 iterations in Python. Statistical significance was established for *p* < 0.05. For multivariate analysis, the Stable Sparse Biomarkers Detection methodology was used, which is based on the combination of resampling techniques and the estimation of a penalized regression to determine quantitative indicators of the degree of stability of the extracted biomarkers. Models were run with a script developed on MATLAB R 2015a. Sparse classifier construction with built-in variable selection was conducted by examining a weighted multivariate linear regression model such as elastic-net (known as Generalized Linear Model Net). The strategy of the resampling method implemented via the perturbation of the data provided random subsamples of subjects, whereas the estimation of a penalized regression allowed the determination of the importance of the variables. The stability indicator was calculated taking into account the principle that if some predictors are significant in all models, they may point to variables that are strong indicators of a stable biomarker. This indicator is the ratio of the number of times the variable is significant in the models for each subsample to the total number of subsamples obtained. The evaluation of performance was based on precision measures obtained from ROC curves. The AUC constitutes an estimate of model efficiency [26].

### 2.8. Statistical Analysis Methods for Predictive Value Estimation

Visual function in terms of pVEP AOz12′ was the predicted outcome of the multivariate models at 12 months. The predictors used in the models’ development were the pre-surgical values of pVEP amplitude, gRNFL and gGCC+IPL thickness, temporal sectors (TS) of RNFL and bi-nasal sectors of GCC+IPL. The models were run as described in the previous section.

## 3. Results

### 3.1. Influence of ESC on the Diagnostic Value of PMA Biomarkers

Forty-two patients with PMA and an equal number of healthy volunteers matched by age were included in the cross-sectional analysis. Electrophysiology data were collected for 35 eyes of PMA patients. The mean latency and AOz of the p100 of pVEP, as well as the *p*-value obtained for the comparison of the six datasets obtained from the most widespread ESC, are summarized in Table 1. All patient groups exhibited significant differences from the healthy control group in terms of the analyzed variables. Significant differences were obtained in the pVEP latency in several comparisons among the datasets at all spatial frequencies. There were also differences in amplitude between RE and LE, RE and WE and BoE and BeE for all spatial frequencies and a few other metrics. As expected, subjects in the control group exhibited good inter-eye evenness (above 0.7, ICC_LOz60′_ = 0.7, ICC _LOz20′_ = 0.7 and ICC _LOz-12′_ = 0.7, ICC_AOz60′_ = 0.75, ICC _AOz20′_ = 0.9 and ICC _AOz12′_ = 0.9), and so RSE was used in the calculations. In PMA patients, the ICC values were ≤ 0.5 (ICC_LOz60′_ = 0.4, ICC _LOz20′_ = 0.4 and ICC _LOz12′_ = 0.2 ICC_AOz60′_ = 0.5, ICC _AOz20′_ = 0.4 and ICC _AOz12′_ = 0.5), which is indicative of poor reliability [25].

Table 2 displays the means and standard deviations of global thickness of RNFL and gGCC+IPL and the sectors of interest. RNFL thickness was the parameter used for classifying eyes as better or worse. Thus, it makes no sense to perform a comparison between such datasets. No differences were found among RE, LE, BoE and RSE datasets regarding average of RNFL, but there were inter-eye differences in the thickness of the temporal sectors. The worse scenario of gGCC+IPL thinning was 57 ± 6 µm, and for bi-nasal sectors, it was 52 ± 6 µm. Furthermore, bi-nasal sector thicknesses were significantly different between the BeE and WE and RE and RSE datasets. Inter-eye correlation was demonstrated for gGCC+IPL and bi-nasal sectors in healthy volunteers (ICC _gRNFL_ = 0.9, ICC_TS_ = 0.8, ICC _gGCC+IPL_ = 0.9, ICC _Bi-NS_ = 0.8), thus RSE was used for comparison. In PMA patients, there was poor inter-eye measurement consistency (ICC _gRNFL_ = 0.4, ICC_TS_ = 0.4, ICC _gGCC+IPL_ = 0.5, ICC _Bi-NS_ = 0.5) [25].

**Figure 1:**
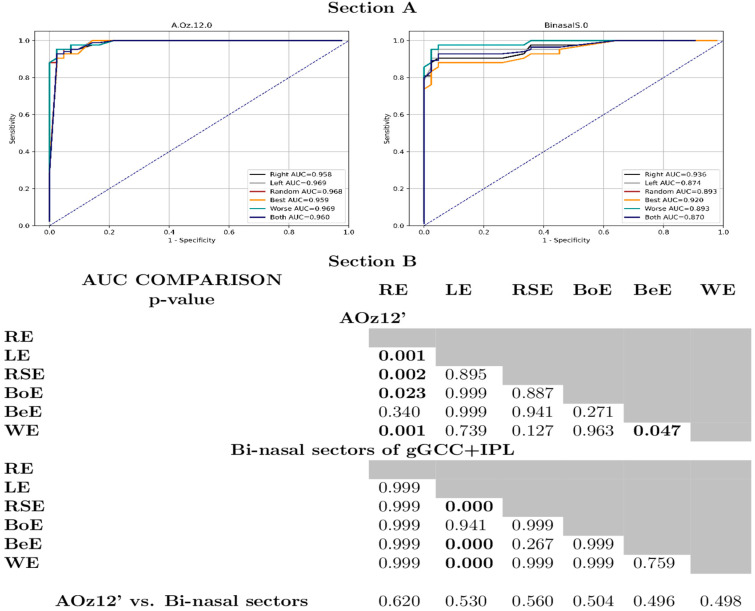
Recipient operating curves of the amplitude of the pVEP and bi-nasal sectors of ganglion cells complex + inner plexiform layer for all datasets. Statistical differences among areas under curves do not have implications for the clinical management of the patients, since all values are high. The amplitudes in Oz12′ and bi-nasal sectors of GCC + IPL using the same ESC for all datasets reveal no differences in the diagnostic values of such biomarkers according to bootstrap analysis with 10,000 iterations. RE: right eye. LE: left eye. RSE: randomly selected eye. BoE: both eyes. BeE: best eye. WE: worst eye. gGCC + IPL: ganglion cells complex + inner plexiform layer. AOz12′: amplitude in Oz12′. AUC: area under curve.

The amplitude of the P100 wave in AOz12′ and the bi-nasal sector thicknesses of GCC+IPL had specificity and sensitivity higher than 0.74. The structural marker exhibited maximal specificity, while the functional one (pVEP) showed maximal sensitivity. Although statistically significant differences in diagnostic performance were observed depending on the ESC, all configurations achieved high AUC values (≥0.87), indicating excellent differentiation between subjects with and without chiasmal compression (Figure 1, Section A, Section B).

### 3.2. Multivariate Analysis

#### 3.2.1. Multivariate Models for PMA Diagnosis

**Figure 2:**
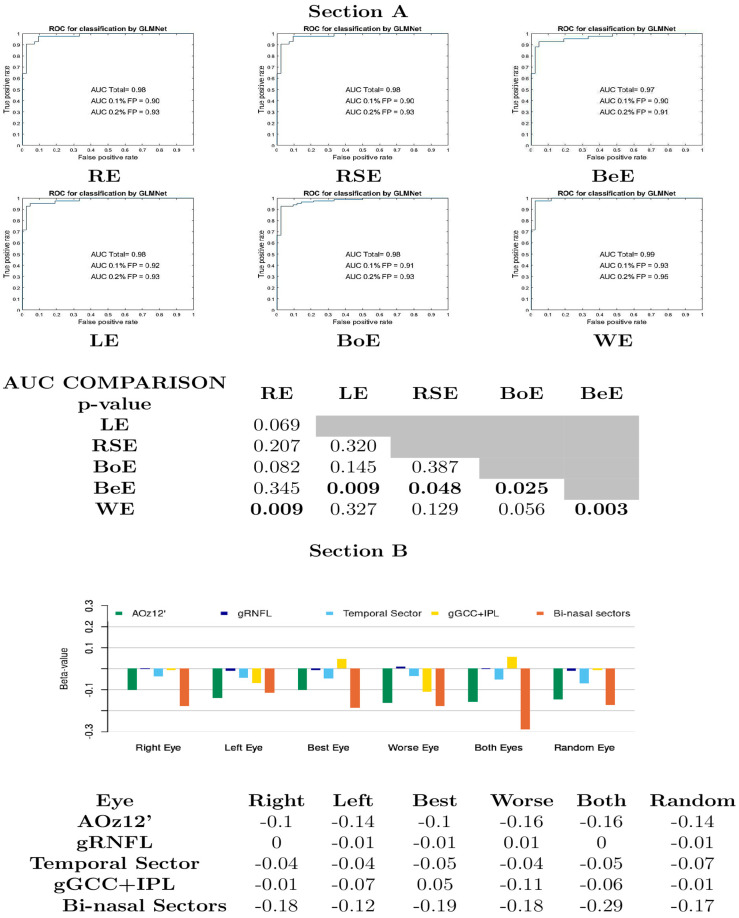
Multivariate model for pituitary macroadenoma diagnosis from pre-surgical datasets with different eye selection criteria. Section A. Recipient operating characteristic curves and bootstrap comparisons of AUCs. Section B. β-values. RE: right eye. LE: left eye. RSE: randomly selected eye. BoE: both eyes. BeE: best eye. WE: worst eye. RNFL: retinal nerve fiber layer. AUC: area under curve.

Multivariate analysis was run for all datasets, including gRNFL, temporal sectors, gGCC+IPL, bi-nasal sector of GCC+IPL thicknesses and pVEP amplitudes as independent variables. A binomial outcome variable (patient or control) was considered as dependent. It is important to highlight that the AUC values of all models are considered high, and among them, WE selection was equal or superior to the rest; additional data are given in Online Resource 1, Section a. In all models, the β values of the proposed biomarkers (bi-nasal sector of GCC+IPL thicknesses and pVEP amplitudes) were higher than those of the rest (Figure 2, section A, section B).

#### 3.2.2. Multivariate Models for PMA Patients’ Follow-Up

**Figure 3:**
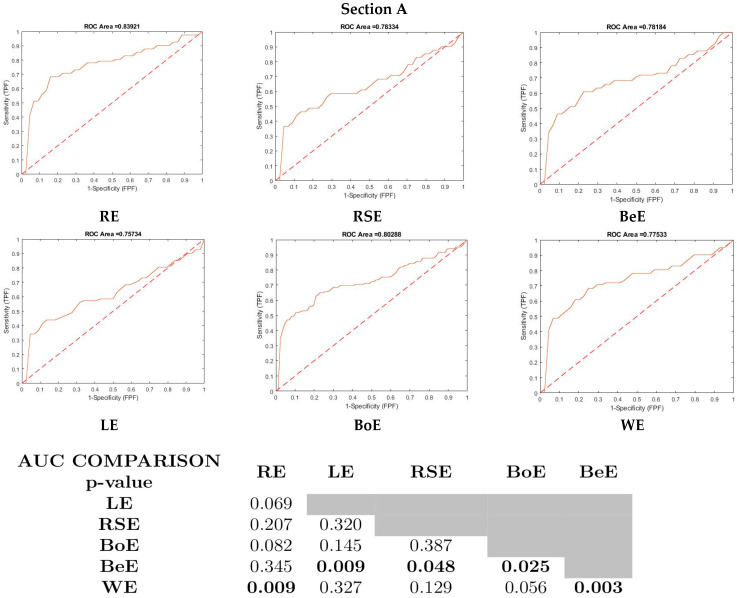

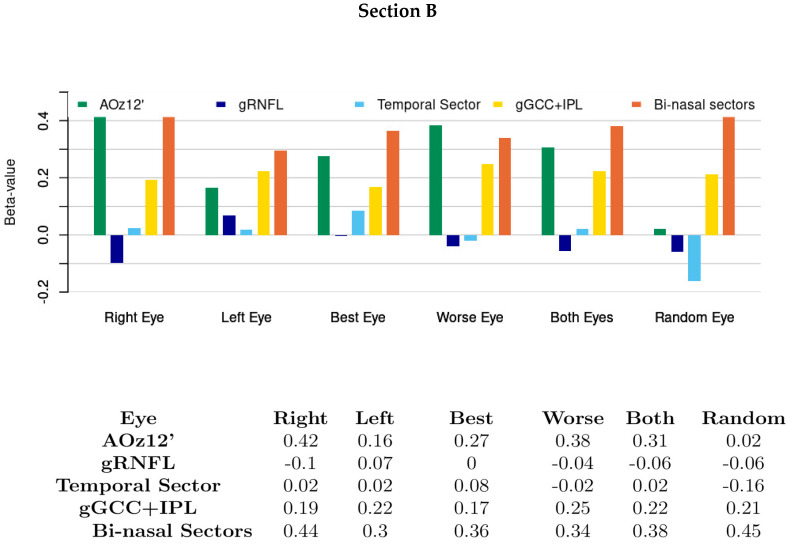
Multivariate model of PMA patients’ post-surgical visual recovery prediction with different ESC datasets. Section a. Recipient operating characteristic curves and bootstrap comparisons of AUC. Section b. β-values. RE: right eye. LE: left eye. RSE: randomly selected eye. BoE: both eyes. BeE: best eye. WE: worst eye. RNFL: retinal nerve fiber layer. AUC: area under curve.

For predictive purposes, amplitudes and latencies at different spatial frequencies (12′, 20′, 60′) were initially evaluated. Ultimately, AOz12′ was included in the multivariate models due to its superior stability and predictive contribution in exploratory analyses. Then, the analysis was conducted for the six datasets post-surgically to estimate AOz12′, using the pre-surgical values of the parameters detailed in the previous section. At three months, models for every ESC with spatial frequencies of pVEP had 0.11 ≤ ICC ≤ 0.71, with a wide CI (0.0; 0.84) and 0.40 ≤ r^2^ ≤ 0.76. The stability of bi-nasal sector thicknesses was 100%, and the stability of AOz decayed from 12′ (range, 83–98%) to 20′and 60′ (range, 56–95%). Hence, the 12-month visual function prediction models received more attention. AOz12′ was the most informative predictor, with 0.78 ≤ ICC ≤ 0.83, narrow CI (0.64; 0.84) and 0.69 ≤ r^2^ ≤ 0.80 vs. AOz20′. AOz60′ exhibited a decreased ICC (0.59 ≤ ICC ≤ 0.82), with a wider CI (0.32; 0.87) and lower r^2^ (0.5 ≤ r^2^ ≤ 0.72) than AOz12′. The predictive values of the different multivariate models according to ESC were the same, with the exception of WE vs. RE and LE (0.76 ≤ AUC ≤ 0.80, Online Resource 2, Section a). The pre-surgical values of the proposed biomarkers had stability ≥ 99%, and the β value ranges were higher than the rest of the parameters—the β-value for the bi-nasal sectors of GCC+IPL was 0.30–0.45, whilst that of AOz12′ was 0.16–0.42—with the exception of the RSE dataset, with a β-value for AOz12′ of 0.02 (Figure 3, Section A, Section B).

## 4. Discussion

### 4.1. ESC’s Influence in the Diagnostic Value of PMA Biomarkers

Various authors have investigated the capacity of OCT and pVEP parameters to be used to detect changes in PMA patients’ visual function and eye structure [27,28,29]. The criteria for selecting the unit of analysis vary widely. In the majority of the cases, BoE are evaluated and added [30,31,32], or the WE is chosen [33,34]. Monteiro et al. used RSE [35], and Agarwal et al. selected RE [36]; Sousa et al. and Sun et al. performed a complex selection process [37,38], while Blanch et al. did not specify theirs [39]. Dataset sizes range from 7 [39] to 114 patients [31], and the number of analyzed eyes from 14 [39] to 210 [32]. All authors have agreed that the thinning of gRNFL and gGCC+IPL is informative of visual pathway compression. Some of them have also described the superiority of gGCC+IPL over gRNFL for diagnosing visual pathway compression with AUC_GCC+IPL_ > 0.85 vs. AUC_RNFL_ (0.73 to 0.79) [16,36]. Some researchers have identified the inferonasal sector (INS) as the most affected [35], and Yum et al. reported an AUC_INS of GCC+IPL_ similar to our findings (AUC_BNS_ = 0.97) [33]. Sousa et al. evaluated the latency and amplitude of multifocal VEP and showed a reduction in amplitude in eyes with visual field defects [37]. Popescu et al. described a delay in latency of P100 in pVEP, even in cases with normal visual fields, as in our study [31].

In the present work, we found a slight variation in AUC values for AOz12′ and bi-nasal GCC+IPL thicknesses from those we reported before [16] due to differences in matching criteria and sample size. In the previous study, matching was performed according to age and sex, while in the present one, matching was performed according to age alone. Univariate analysis showed that WE is the best choice as ESC. The values of AOz12′ and the thickness of bi-nasal sectors of GCC+IPL, under all criteria, remain high (AUC > 0.87) and equivalent. Furthermore, PMA diagnosis multivariate models have demonstrated that both biomarkers had the highest stability, interclass correlation, rho and β-values, while the maximum AUCs were achieved under WE dataset analysis (AUC > 0.97).

### 4.2. Follow-Up and Visual Function Prediction

Although several prognostic factors affecting visual outcome after sellar and parasellelar tumor resection have been investigated [7,8,9,40,41,42], the prediction of the postoperative visual recovery of pituitary adenoma patients by applying feasible and accurate approaches remains challenging [10]. Postoperative periods for outcome evaluation have ranged from a few weeks to several months. OCT analysis has been the most widely used, although few authors have relied on electrophysiological biomarkers. For visual function prediction, BeE is most commonly used as ESC with or without checking inter-eye correlation [1,11,12,13,14,16,30,32,36]. However, some authors have not specified the selected eye [2]. Among structural parameters, gRNFL and gGCC+IPL thickness measurements have exhibited moderate to high associations with the median deviation of the visual field and visual acuity. Superior nasal sector measurements of GCC+IPL thickness were found to be the most accurate by Tieger et al. [30]. In the present work, the AUC values of multivariate models exceed 0.77. Note that for five of the models, the proposed biomarkers are the most informative with regard to visual pathway function restoration. Regardless, the bi-nasal sector of GCC+IPL thickness appears to be the most stable marker and a strong indicator of post-surgical success. In addition, multivariate analysis has confirmed that 12 months is a suitable period for monitoring visual restoration and emphasizes that AOz12′ and bi-nasal sectors of GCC+IPL are robust predictive biomarkfers for PMA follow-up. This approach provides a practical and accurate method for predicting visual outcomes in patients with PMA.

Predictive biomarkers of PMA post-surgical function recovery have also been assessed using parameters derived from blood and image analysis. Guan X et al. developed a multivariate model based on the contributions of the products of eight genes (*BMP6, CIB2, FABP5*, *HOMER2*, *MAML3*, *NIN*, *PRKG2* and *SIDT2*) associated with the immune response and non-functional pituitary neuroendocrine tumor invasion (Knosp scale) and showed that this model’s prediction accuracy was mosderate (AUC = 0.671) [43]. Other authors have developed models and markers based on medical imaging. Hisanaga et al. proposed that the optical nerve kinking angle is an indicator of the good post-operative improvement of visual acuity and the visual field when cut-offs are 102.5° and 114.5°, respectively [44], based on contrast-enhanced, fast MRI steady-state acquisition data. Likewise, Shinohara et al. showed that the optic nerve canal bending angle may be associated with pre-surgical GCC+IPL thickness and described how this may be associated with visual restoration after surgery in terms of best-corrected visual acuity [45]. In addition, Zhang et al. proposed a model based on the delta-radiomics of the optic chiasm to predict the 12-month post-operative visual recovery of PMA patients who underwent endoscopic endonasal transsphenoidal surgery. Delta-radiomics of the optic chiasm was calculated based on features extracted from coronal T2-weighted images, followed by machine learning modeling. Chiasmal thickness, chiasmal deformed angle (in degrees) and chiasmal suprasellar extension (mm), age, gender, histological subtype and ki67 index were the parameters considered, and an AUC = 0.840 was obtained for an independent test set [46]. Such biomarker evaluation requires more expensive technologies.

## 5. Conclusions

The diagnostic values of the bi-nasal sector of the ganglion cell complex’s thickness and the amplitude in Oz at 12′ of the pattern visual evoked potential are affected by eye selection criteria, but both biomarkers exhibit equivalently high accuracy and stability for the same criteria. Univariate analysis and multivariate models highlight that the worse eye is the best choice for evaluation because the maximum AUC is achieved with this data subset. The pre-surgical values of both biomarkers provide a robust prediction of 12-months post-surgical visual function recovery, but further studies are needed to validate these findings.

## Figures and Tables

**Table 1:** Descriptive statistics of latency and amplitude pattern visual evoked potentials. Latencies and amplitude means compared according to eye selection criteria. Kruskal–Wallis analysis plus Holm-adjusted Wilcoxon test. *p*-values of the comparisons according to eye selection criteria are displayed for the pituitary macroadenoma group.

**Parameter**	**Right** **Eye** **N = 19**	**Left** **Eye** **N = 16**	**Randomly** **Selected Eye** **N = 18**	**Both Eyes** **N = 35**	**Best Eye** **N = 19**	**Worst Eye** **N = 19**	**Healthy Volunteers** **N = 42**
	**Mean ± SD**	**Mean ± SD**	**Mean ± SD**	**Mean ± SD**	**Mean ± SD**	**Mean ± SD**	**Mean ± SD**
**LOz60′**	120 ± 17	117 ± 12	120 ± 17	119 ± 15	117 ± 16	121 ± 13	107 ± 4
***p*-value PMAp. vs. HV**	9.6 × 10^−12^	9.6 × 10^−12^	9.6 × 10^−12^	1.9 × 10^−15^	9.6 × 10^−12^	9.6 × 10^−12^	
**LOz20′**	122 ± 8	123 ± 9	124 ± 7	123 ± 9	122 ± 9	124 ± 9	111 ± 7
***p*-value PMAp. vs. HV**	4.4 × 10^−11^	5.6 × 10^−11^	1.1 × 10^−11^	4.3 × 10^−14^	1.2 × 10^−10^	4.0 × 10^−11^	
**LOz12′**	125 ± 8	127 ± 10	125 ± 9	124 ± 9	127 ± 9	126 ± 9	119 ± 11
***p*-value PMAp. vs. HV**	1.3 × 10^−7^	1.0 × 10^−7^	1.3 × 10^−8^	1.1 × 10^−9^	7.6 × 10^−8^	2.7 × 10^−7^	
***p*-value LOz60′**	**RE**	**LE**	**RSE**	**BoE**	**BeE**		
RE							
LE	**0.000**						
RSE	0.454	**0.000**					
BoE	0.217	**0.008**	**0.008**				
BeE	**0.001**	0.618	**0.000**	**0.034**			
WE	**0.016**	**0.000**	0.216	**0.000**	**0.000**		
***p*-value LOz20′**	RE	LE	RSE	BoE	BeE		
RE							
LE	0.165						
RSE	**0.011**	0.963					
BoE	0.881	0.729	0.087				
BeE	0.963	**0.049**	**0.002**	0.450			
WE	**0.001**	0.450	0.963	**0.004**	**0.000**		
***p*-value LOz12′**	RE	LE	RSE	BoE	BeE		
RE							
LE	0.070						
RSE	0.830	**0.003**					
BoE	0.624	0.496	0.070				
BeE	**0.024**	0.916	**0.001**	0.250			
WE	0.407	**0.000**	0.916	**0.005**	**0.000**		
**Parameter**	**RE** **N = 19**	**LE** **N = 16**	**RSE** **N = 18**	**BoE** **N = 35**	**BeE** **N = 19**	**WE** **N = 19**	**Healthy Volunteers** **N = 42**
	**Mean ± SD**	**Mean ± SD**	**Mean ± SD**	**Mean ± SD**	**Mean ± SD**	**Mean ± SD**	**Mean ± SD**
**AOz60′**	5.04 ± 2.32	4.69 ± 1.71	4.71 ± 1.90	4.88 ± 2.03	5.10 ± 2.26	4.58 ± 1.78	15.71 ± 3.54
***p*-value PMAp. vs. HV**	3.3 × 10^−14^	1.6 × 10^−14^	4.1 × 10^−14^	1.9 × 10^−18^	4.8 × 10^−14^	2.6 × 10^−14^	
**AOz20′**	5.00 ± 2.71	4.28 ± 2.08	4.53 ± 2.36	4.67 ± 2.41	4.93 ± 2.62	4.32 ± 2.20	17.08 ± 5.01
***p*-value PMAp. vs. HV**	3.9 × 10^−15^	1.6 × 10^−15^	3.7 × 10^−15^	1.0 × 10^−19^	5.5 × 10^−15^	2.9 × 10^−15^	
**AOz12′**	4.33 ± 2.64	3.98 ± 2.15	4.05 ± 2.23	4.17 ± 2.40	4.41 ± 2.58	3.85 ± 2.22	17.83 ± 7.72
***p*-value PMAp. vs. HV**	5.7 × 10^−15^	2.9 × 10^−15^	1.0 × 10^−14^	1.9 × 10^−19^	1.1 × 10^−14^	3.8 × 10^−15^	
***p*-value AOz60′**	RE	LE	RSE	BoE	BeE		
RE							
LE	**0.008**						
RSE	0.057	0.899					
BoE	0.706	0.072	0.293				
BeE	0.899	**0.001**	**0.006**	0.232			
WE	**0.000**	0.705	0.293	**0.000**	**0.000**		
***p*-value AOz20′**	RE	LE	RSE	BoE	BeE		
RE							
LE	**0.000**						
RSE	**0.012**	0.137					
BoE	0.149	**0.000**	0.735				
BeE	0.853	**0.000**	0.171	0.853			
WE	**00.00**	0.853	0.853	**0.037**	**0.007**		
***p*-value AOz12′**	RE	LE	RSE	BoE	BeE		
RE							
LE	**0.005**						
RSE	0.120	0.787					
BoE	0.787	0.062	0.787				
BeE	0.787	**0.001**	**0.035**	0.292			
WE	**0.000**	0.787	0.211	**0.001**	**0.000**		

RE: right eye. LE: left eye. RSE: randomly selected eye. BoE: both eyes. BeE: best eye. WE: worst eye. LOz12′: latency of visual evoked potential in Oz 12′. AOz12′: amplitude of visual evoked potential in Oz 12′. Number in bold means ‘*p* < 0.05’. Variations in sample size can be explained by the fact that not all the subjects attended every designated consultation and not every eye was suitable for assessment due to the requisites of the tests performed. The 42 healthy volunteers were selected using propensity score matching by age and selecting 42 eyes randomly (one eye per subject) with the same python code as was used for ‘randomly selected eye’ in the patient group since there was high interclass correlation between right and left eyes in these healthy subjects, as expected. The parameters for each group were also statistically different from those of healthy controls. PMA patients (PMAp) vs. healthy volunteers (HV) comparisons were performed using the Mann–Whitney test.

**Table 2:** Thickness of global retinal nerve fiber layer and ganglion cell complex plus inner plexiform layer. Mean comparisons according to eye selection criteria. Kruskal–Wallis analysis plus Holm-adjusted Wilcoxon test. *p*-values of the comparisons according to eye selection criteria are displayed in the pituitary macroadenoma group.

**Parameter**	**Right Eye** **N = 28**	**Left Eye** **N = 28**	**Randomly** **Selected** **Eye** **N = 28**	**Both** **Eyes** **N = 56**	**Best** **Eye** **N = 28**	**Worst Eye** **N = 28**	**Healthy Volunteers** **N = 42**
	**Mean ± SD**	**Mean ± SD**	**Mean ± SD**	**Mean ± SD**	**Mean ± SD**	**Mean ± SD**	**Mean ± SD**
**gRNFL**	81 ± 11	80 ± 11	79 ± 11	81 ± 11	84 ± 10	77 ± 11	101 ± 11
***p*-value PMAp. vs. HV**	3.8 × 10^−11^	1.6 × 10^−11^	1.2 × 10^−11^	1.2 × 10^−14^	2.3 × 10^−10^	2.4 × 10^−12^	
**Temporal Sectors**	52 ± 8	48 ± 8	48 ± 8	50 ± 8	50 ± 8	50 ± 8	64 ± 10
***p*-value PMAp. vs. HV**	2.9 × 10^−8^	1.6 × 10^−11^	6.3 × 10^−9^	1.2 × 10^−12^	2.3 × 10^−9^	2.7 × 10^−10^	
***p*-value gRNFL**	RE	LE	RSE	BoE	BeE	WE	
RE							
LE	0.884						
RSE	0.330	0.999					
BoE	0.999	0.999	0.856				
***p*-value** **Temporal Sectors**	RE	LE	RSE				
RE							
LE	**0.018**						
RSE	**0.014**	0.999					
BoE	0.558	0.509	0.491				
**Parameter**	**RE**	**LE**	**RSE**	**BoE**	**BeE**	**WE**	**Healthy Volunteers**
	**Mean ± SD**	**Mean ± SD**	**Mean ± SD**	**Mean ± SD**	**Mean ± SD**	**Mean ± SD**	**Mean ± SD**
gGCC+IPL	68 ± 9	65 ± 9	64 ± 8	67 ± 9	70 ± 9	63 ± 9	85 ± 5
***p*-value PMAp. vs. HV**	1.9 × 10^−12^	6.2 × 10^−13^	1.2 × 10^−12^	1.9 × 10^−16^	2.1 × 10^−11^	5.1 × 10^−14^	
Bi-nasal sectors	63 ± 12	60 ± 13	58 ± 11	61 ± 12	64 ± 13	58 ± 11	87 ± 6
***p*-value PMAp. vs. HV**	2.8 × 10^−13^	6.1 × 10^−14^	2.0 × 10^−13^	1.1 × 10^−17^	1.5 × 10^−12^	1.0 × 10^−14^	
***p*-value gGCC+IPL**	RE	LE	RSE	BoE	BeE		
RE							
LE	0.399						
RSE	0.143	0.999					
BoE	0.999	0.999	0.713				
BeE	0.999	0.051	**0.010**	0.200			
WE	**0.019**	0.999	0.999	0.143	**0.001**		
***p*-value** **Bi-nasal sectors**	RE	LE	RSE	BoE	BeE		
RE							
LE	0.381						
RSE	**0.041**	0.999					
BoE	0.999	0.999	0.289				
BeE	0.999	0.122	**0.008**	0.536			
WE	0.120	0.999	0.999	0.564	**0.029**		

RE: right eye. LE: left eye. RSE: randomly selected eye. BoE: both eyes. BeE: best eye. WE: worst eye. gRNFL: global retinal nervous fiber layer. gGCC+IPL: global ganglion cell complex + inner plexiform layer. Variations in sample size can be explained by the fact that not all the subjects attended every designed consultation, and not every eye was suitable for assessment due to the requisites of the tests performed. The 42 healthy volunteers were selected using propensity score matching by age and selecting 42 eyes randomly (one eye per subject) with the same python code as used for ‘randomly selected eye’ in the patient group, since there was a high interclass correlation between right and left eyes in these healthy subjects, as expected. PMA patients (PMAp) vs. healthy volunteers (HV) were compared using Mann–Whitney test.

## Data Availability

If anyone is interesting in raw data supporting these findings do not hesitate to contact the authors, we will kindly collaborate.

## References

[1] Ying G.-S., Maguire M.G., Glynn R.J., Rosner B. (2020). Calculating sensitivity, specificity, and predictive values for correlated eye data. Investig. Ophthalmol. Vis. Sci..

[2] Dong R., Ying G.-S. (2023). Characteristics of Design and Analysis of Ophthalmic Randomized Controlled Trials: A Review of Ophthalmic Papers 2020–2021. Ophthalmol. Sci..

[3] Bunce C., Patel K.V., Xing W., Freemantle N., Doré C.J., Ophthalmology O.S. (2014). Ophthalmic statistics note 1: Unit of analysis. Br. J. Ophthalmol..

[4] Ji X., Zhuang X., Yang S., Zhang K., Li X., Yuan K., Zhang X., Sun X. (2023). Visual field improvement after endoscopic transsphenoidal surgery in patients with pituitary adenoma. Front. Oncol..

[5] Karakosta A., Vassilaki M., Plainis S., Elfadl N.H., Tsilimbaris M., Moschandreas J.J. (2012). Choice of analytic approach for eye-specific outcomes: One eye or two?. Am. J. Ophthalmol..

[6] Armstrong R.A. (2013). Statistical guidelines for the analysis of data obtained from one or both eyes. Ophthalmic Physiol. Opt..

[7] Oray M., Önal S., Akbay A.K., Tutkun İ.T. (2017). Diverse clinical signs of ocular involvement in cat scratch disease. Turk. J. Ophthalmol..

[8] Varela M.D., Conti G.M., Malka S., Vaclavik V., Mahroo O.A., Webster A.R., Tran V., Michaelides M. (2023). Coats-like Vasculopathy in Inherited Retinal Disease: Prevalence, Characteristics, Genetics, and Management. Ophthalmology.

[9] Murdoch I.E., Morris S.S., Cousens S.N. (1998). People and eyes: Statistical approaches in ophthalmology. Br. J. Ophthalmol..

[10] Kumari R. (2024). Senile Cataract. J. Community Med..

[11] Distelhorst J.S., Hughes G.M. (2003). Open-angle glaucoma. Am. Fam. Physician.

[12] Lambertus S., Bax N.M., Groenewoud J.M., Cremers F.P., van der Wilt G.J., Klevering B.J., Theelen T., Hoyng C.B. (2016). Asymmetric inter-eye progression in Stargardt disease. Investig. Ophthalmol. Vis. Sci..

[13] Jauregui R., Chan L., Oh J.K., Cho A., Sparrow J.R., Tsang S.H. (2020). Disease asymmetry and hyperautofluorescent ring shape in retinitis pigmentosa patients. Sci. Rep..

[14] KesKın A.O., İdıman F., Kaya D., Bircan B. (2020). Idiopathic intracranial hypertension: Etiological factors, clinical features, and prognosis. Noro Psikiyatr. Arsivi.

[15] Ying G.-S., Maguire M.G., Glynn R., Rosner B. (2018). Tutorial on biostatistics: Statistical analysis for correlated binary eye data. Ophthalmic Epidemiol..

[16] Hernández-Echevarría O., Cuétara-Lugo E.B., Pérez-Benítez M.J., González-Gómez J.C., González-Diez H.R., Mendoza-Santiesteban C.E. (2022). Bi-nasal sectors of ganglion cells complex and visual evoked potential amplitudes as biomarkers in pituitary macroadenoma management. Front. Integr. Neurosci..

[17] General assembly of the world medical association (2014). World Medical Association Declaration of Helsinki: Ethical principles for medical research involving human subjects. J. Am. Coll. Dent..

[18] Racette L., Fischer M., Bebie H., Holló G., Johnson C.A., Matsumoto C.J. (2016). ; Visual Field Digest.

[19] Walsh F.B., Hoyt W.F. (2008). Walsh and Hoyt’s Clinical Neuro-Ophthalmology: The Essentials.

[20] Odom J.V., Bach M., Brigell M., Holder G.E., McCulloch D.L., Mizota A., Tormene A.P., International Society for Clinical Electrophysiology of Vision (2016). ISCEV standard for clinical visual evoked potentials: (2016 update). Doc. Ophthalmol..

[21] Hamilton R., Bach M., Heinrich S.P., Hoffmann M.B., Odom J.V., McCulloch D.L., Thompson D.A. (2021). ISCEV extended protocol for VEP methods of estimation of visual acuity. Doc. Ophthalmol..

[22] Robson A.G., Nilsson J., Li S., Jalali S., Fulton A.B., Tormene A.P., Holder G.E., Brodie S.E. (2018). ISCEV guide to visual electrodiagnostic procedures. Doc. Ophthalmol..

[23] Gadelha M.R., Barbosa M.A., Lamback E.B., Wildemberg L.E., Kasuki L., Ventura N. (2022). Pituitary MRI standard and advanced sequences: Role in the diagnosis and characterization of pituitary adenomas. J. Clin. Endocrinol. Metab..

[24] Daly A.F., Beckers A. (2020). The Epidemiology of Pituitary Adenomas. Endocrinol. Metab. Clin. N. Am..

[25] Koo T.K., Li M.Y. (2016). A guideline of selecting reporting intraclass correlation coefficients for reliability research. J. Chiropr. Med..

[26] Bosch-Bayard J., Galán-García L., Fernandez T., Lirio R.B., Bringas-Vega M.L., Roca-Stappung M., Ricardo-Garcell J., Harmony T., Valdes-Sosa P.A. (2018). Stable sparse classifiers identify qEEG signatures that predict learning disabilities (NOS) severity. Front. Neurosci..

[27] Lachowicz E., Lubiński W., Gosławski W., Andrysiak-Mamos E., Kaźmierczyk-Puchalska A., Syrenicz A.J. (2021). The electrophysiological tests in the early detection of visual pathway dysfunction in patients with microadenoma. Doc. Ophthalmol..

[28] Nikoobakht M., Pourmahmoudian M., Nekoo Z.A., Rahimi S., Arabi A.R., Shirvani M., Koohestani H. (2022). The role of optical coherence tomography in early detection of retinal nerve fiber layer damage in pituitary adenoma. Maedica.

[29] Jeong S.S., Funari A., Agarwal V.J.W.N. (2022). Diagnostic and prognostic utility of optical coherence tomography in patients with sellar/suprasellar lesions with chiasm impingement: A systematic review/meta-analyses. World Neurosurg..

[30] Tieger M.G., Hedges T.R., Ho J., Erlich-Malona N.K., Vuong L.N., Athappilly G.K., Mendoza-Santiesteban C.E. (2017). Ganglion cell complex loss in chiasmal compression by brain tumors. J. Neuroophthalmol..

[31] Popescu M., Carsote M., Popescu I.A.S., Costache A., Ghenea A., Turculeanu A., Singer C.E., Iana O., Ungureanu A. (2021). The role of the visual evoked potential in diagnosing and monitoring pituitary adenomas. Res. Sci. Today.

[32] Ergen A., Ergen S.K., Gunduz B., Subasi S., Caklili M., Cabuk B., Anik I., Ceylan S. (2023). Retinal vascular and structural recovery analysis by optical coherence tomography angiography after endoscopic decompression in sellar/parasellar tumors. Sci. Rep..

[33] Yum H.R., Park S.H., Park H.-Y.L., Shin S.Y.J.P.O. (2016). Macular ganglion cell analysis determined by cirrus HD optical coherence tomography for early detecting chiasmal compression. PLoS ONE.

[34] Lee G.-I., Park K.-A., Oh S.Y., Kong D.-S.J.S.R. (2020). Analysis of optic chiasmal compression caused by brain tumors using optical coherence tomography angiography. Sci. Rep..

[35] Monteiro M.L., Hokazono K., Fernandes D.B., Costa-Cunha L.V., Sousa R.M., Raza A.S., Wang D.L., Hood D.C. (2014). Evaluation of inner retinal layers in eyes with temporal hemianopic visual loss from chiasmal compression using optical coherence tomography. Investig. Ophthalmol. Vis. Sci..

[36] Agarwal R., Jain V.K., Singh S., Charlotte A., Kanaujia V., Mishra P., Sharma K. (2021). Segmented retinal analysis in pituitary adenoma with chiasmal compression: A prospective comparative study. Indian J. Ophthalmol..

[37] Sousa R.M., Oyamada M.K., Cunha L.P., Monteiro M.L.J.I.O., Science V. (2017). Multifocal visual evoked potential in eyes with temporal hemianopia from chiasmal compression: Correlation with standard automated perimetry and OCT findings. Investig. Ophthalmol. Vis. Sci..

[38] Sun M., Zhang Z., Ma C., Chen S., Chen X. (2017). Quantitative analysis of retinal layers on three-dimensional spectral-domain optical coherence tomography for pituitary adenoma. PLoS ONE.

[39] Blanch R.J., Micieli J.A., Oyesiku N.M., Newman N.J., Biousse V. (2018). Optical coherence tomography retinal ganglion cell complex analysis for the detection of early chiasmal compression. Pituitary.

[40] Park S.H., Kang M.S., Kim S.Y., Lee J.-E., Shin J.H., Choi H., Kim S.J. (2021). Analysis of factors affecting visual field recovery following surgery for pituitary adenoma. Int. Ophthalmol..

[41] Qiao N., Ma Y., Chen X., Ye Z., Ye H., Zhang Z., Wang Y., Lu Z., Wang Z., Xiao Y. (2022). Machine learning prediction of visual outcome after surgical decompression of sellar region tumors. J. Pers. Med..

[42] Anik I., Anik Y., Koc K., Ceylan S., Genc H., Altintas O., Ozdamar D., Ceylan D.B. (2011). Evaluation of early visual recovery in pituitary macroadenomas after endoscopic endonasal transphenoidal surgery: Quantitative assessment with diffusion tensor imaging (DTI). Acta Neurochir..

[43] Guan X., Wang Y., Zhang C., Ma S., Zhou W., Jia G., Jia W. (2022). Surgical Experience of Transcranial Approaches to Large-to-Giant Pituitary Adenomas in Knosp Grade 4. Front. Endocrinol..

[44] Hisanaga S., Kakeda S., Yamamoto J., Watanabe K., Moriya J., Nagata T., Fujino Y., Kondo H., Nishizawa S., Korogi Y. (2017). Pituitary macroadenoma and visual impairment: Postoperative outcome prediction with contrast-enhanced FIESTA. Am. J. Neuroradiol..

[45] Shinohara Y., Todokoro D., Yamaguchi R., Tosaka M., Yoshimoto Y., Akiyama H. (2022). Retinal ganglion cell analysis in patients with sellar and suprasellar tumors with sagittal bending of the optic nerve. Sci. Rep..

[46] Zhang Y., Zheng J., Huang Z., Teng Y., Chen C., Xu J. (2023). Predicting visual recovery in pituitary adenoma patients post-endoscopic endonasal transsphenoidal surgery: Harnessing delta-radiomics of the optic chiasm from MRI. Eur. Radiol..

